# Integrative analysis in head and neck cancer reveals distinct role of miRNome and methylome as tumour epigenetic drivers

**DOI:** 10.1038/s41598-024-59312-z

**Published:** 2024-04-20

**Authors:** Katarina Mandić, Nina Milutin Gašperov, Ksenija Božinović, Emil Dediol, Jure Krasić, Nino Sinčić, Magdalena Grce, Ivan Sabol, Anja Barešić

**Affiliations:** 1 Division of Electronics, Ruđer Bošković Institute, Zagreb, Croatia; 2 Division of Molecular Medicine, Ruđer Bošković Institute, Zagreb, Croatia; 3 Department of Maxillofacial Surgery, Clinical Hospital Dubrava, Zagreb, Croatia; 4 Department of Medical Biology, University of Zagreb School of Medicine, Zagreb, Croatia; 5 Centre of Excellence in Reproductive and Regenerative Medicine, University of Zagreb School of Medicine, Zagreb, Croatia; 6 Biomedical Research Centre Šalata, University of Zagreb School of Medicine, Zagreb, Croatia

**Keywords:** Head and neck cancer, HPV, miRNome, Methylome, Transcriptome, Integrative analysis, Data integration, Head and neck cancer, Cancer epigenetics

## Abstract

Head and neck cancer is the sixth most common malignancy worldwide, with the relatively low 5-year survival rate, mainly because it is diagnosed at a late stage. Infection with HPV is a well known aetiology, which affects the nature of these cancers and patients’ survival. Besides, it is considered that the main driving force for this type of cancer could be epigenetics. In this study we aimed to find potential epigenetic biomarkers, by integrating miRNome, methylome, and transcriptome analyses. From the fresh head and neck cancer tissue samples, we chose a group for miRNome, methylome and transcriptome profiling, in comparison to adequate control samples. Bioinformatics analyses are performed in R v4.2.2. Count normalisation and group differential expression for mRNA and the previously obtained miRNA count data was performed with DESeq2 v1.36. Gene set enrichment analysis was performed and visualised using gProfiler2 v0.2.1 Identification of miRNA targets was performed by querying in miRTarBase using multiMiR v1.18.0. Annotation of CpG sites merging into islands was obtained from RnBeads.hg19 v1.28.0. package. For the integrative analysis we performed kmeans clustering using stats v4.2.2 package, using 8–12 clusters and nstart 100. We found that transcriptome analysis divides samples into cancers and controls clusters, with no relation to HPV status or cancer anatomical location. Differentially expressed genes (n = 2781) were predominantly associated with signalling pathways of tumour progression. We identified a cluster of genes under the control of the transcription factor *E2F* that are significantly underexpressed in cancer tissue, as well as T cell immunity genes and genes related to regulation of transcription. Among overexpressed genes in tumours we found those that belong to cell cycle regulation and vasculature. A small number of genes were found significantly differentially expressed in HPV-positive *versus* HPV-negative tumours (for example *NEFH*, *ZFR2*, *TAF7L*, *ZNF541*, and *TYMS*). In this comprehensive study on an overlapping set of samples where the integration of miRNome, methylome and transcriptome analysis were performed for head and neck cancer, we demonstrated that the majority of genes were associated exclusively with miRNome or methylome and, to a lesser extent, under the control of both epigenetic mechanisms.

## Introduction

Head and neck cancer is the sixth most common malignancy worldwide, with head and neck squamous cell carcinoma (HNSCC) accounting for more than 90% of cases. Unfortunately, HNSCC often gets diagnosed in a late phase when it is challenging to treat the tumour, which contributes to a poor 5-year survival rate, currently at 66%^1^. Even though this type of cancer arises from a single cell type, i.e. the squamous cell, HNSCC is surprisingly of very heterogeneous nature^2^. Primary site of tumour origin contributes to tumour heterogeneity, with most common sites being oral cavity, oropharynx, larynx, and sinonasal tract^3^. Besides tumour location, the infection with high-risk human papilloma virus (HPV) types, especially in oropharyngeal area predominantly affects HNSCC nature and patients’ survival^4^. HPV-positive and HPV-negative HNSCC can substantially differ in terms of aetiology, genetics and epigenetics^1,5^. HPV-positive HNSCC mostly affects the younger population, with no or low levels of smoking and/or alcohol use^6^. Moreover, unlike the HPV-negative group, preferable site of tumour origin for HPV-positive HNSCC is oropharynx and it has been reported in many Western countries that this group of patients is associated with better overall survival and better response to therapy^7^. There has been a significant shift in the incidence of HPV-positive oropharyngeal squamous cancer in Western countries, where HPV is associated to HNSCC in almost 70% of cases^8^, and in fact, increase in 5-year survival of overall HNSCC cases could be contributed to the higher prevalence of HPV-associated HNSCC^1^.

Up to date, many studies reported better outcomes for HPV-positive HNSCC^9–11^, however, the consensus on specific biomarkers, which could improve diagnostic, prognostic and/or therapeutic approaches is still missing. Lack of consensus biomarkers could partially be owed to differences in studied populations, sometimes inadequately registered tumour site of origin, and possibly to the lack of stratification by the HPV status. Therefore, we consider the cohort presented below to be a specific case of tumours in terms of lifestyle, i.e. higher smoking and alcohol intake rates.

In this study, we aimed to identify epigenetic patterns in HNSCC since the main driving force for the HNSCC development is considered to be epigenetics, rather than genetics^12^. We have previously analysed the content and transcriptional levels of whole genome microRNA (miRNA) profile (miRNome) in HPV-positive and HPV-negative oral and oropharyngeal HNSCC samples using next generation sequencing and validated with qRT-PCR^13^. We have also analysed the whole genome methylation profile (methylome) in the same set of samples using the whole genome methylation array and validated by pyrosequencing^14^. In this study, we assessed the whole genome transcriptome analysis on the same sample set and combined the mRNA, miRNA and methylation information to elucidate relevant epigenetic mechanisms and their interactions. Motivated by the limited literature regarding integrative studies on epigenetic changes, we set out to elucidate whether miRNAs or DNA methylation have a more prominent impact on HNSCC development. Moreover, we aimed to identify key mechanisms and/or cellular signalling pathways of genes that have been differentially expressed in HNSCC compared to healthy controls. Consequently, we provide a better understanding of the epigenetic influence on the development and progression of HPV-positive and HPV-negative HNSCC, shedding light on mechanisms underlying prognostic, diagnostic and therapeutic strategies. In addition, integrative analysis points out key target genes and signalling pathways deregulated in HNSCC in Croatian and similar populations.

## Material and methods

### Patient material

**Figure 1:**
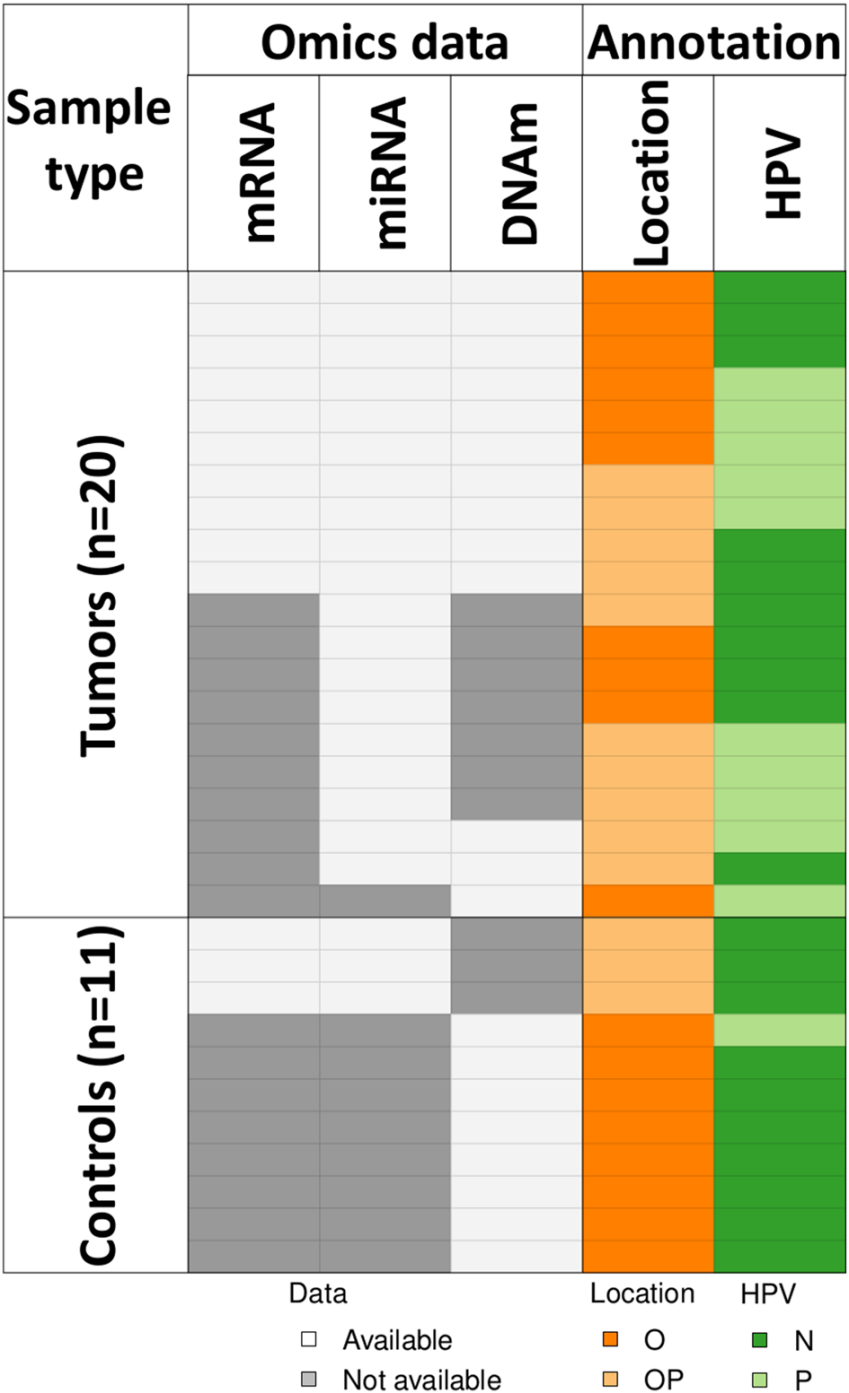
Samples used in integrative the analysis. Sequenced samples are shown in white. O, oral; OP, oropharyngeal; P, HPV-positive; N, HPV-negative.

A total of 61 fresh oropharyngeal (OP) and oral (O) HNSCC primary tumour samples have been obtained from Dubrava Clinical Hospital in the 2015–2018 time period. Twenty-two of them and three non-malignant tonsil control samples were included in the miRNA profiling^13^, column 2 in Fig. 1. Of 22 sequenced samples, two were excluded, due to one sample being a recurrent tumour and one outlier. miRNome analysis and detailed patients’ socioepidemiologic and clinical data have been described in our previous study. From the previously published analysis of HNSCC methylome^14^, of 22 samples included in miRNA study, 12 HNSCC samples were included in the whole genome methylation profiling (column 3, Fig. 1), as well as one additional sample that had methylome, but no transcriptome and miRNome profile. Eight healthy swabs of oral mucosa were included in the methylome profiling, which served as control samples.

For the transcriptome profiling, we selected 10 fresh HNSCC samples (6 oral and 4 oropharyngeal) with previous miRNome and methylome data (column 1, Fig. 1). Three fresh tonsil tissues from the miRNome dataset were included in the transcriptome profiling as control tissue. HPV status was assessed by PCR in all samples, as described previously^15^.

### Transcriptome profiling

As stated above, 13 samples were selected for transcriptome analysis in the current study to complement previously obtained omics results. Sample annotation used in transcriptome analysis and subsequent integrative epigenetic study is presented in Fig. 1. Transcriptome was assessed using TrueSeq Stranded mRNA (Illumina) kit for library preparation, followed by sequencing on Nextseq500 instrument using NextSeq HIGH 150 cycles (Illumina) reagents on high output flow cell in paired end mode. Debarcoding was performed using Basespace (Illumina) platform. Quality control of sequencing data was performed with FastQC v0.11.9. Sequences were both aligned to the human reference transcriptome (GRCh37 downloaded from GENCODE, released in 2013) and quantified using salmon v1.5.0 with default parameters^16^, reporting estimated fragment counts per transcript. An average of 25.2 ± 2.2 million fragments were reported per sample (Supplemental Table S1).

### Bioinformatics preprocessing

All bioinformatics analyses listed hereafter were performed in R v4.2.2, and refer to R language packages, unless otherwise stated. The preprocessing consisted of transcriptome gene count determination, reprocessing of miRNA counts (for a subset of samples from Božinović et al.^13^) and reprocessing of differentially methylated CpGs (for a subset of samples from Milutin Gašperov et al.^14^), here expanded to CpG islands.

Count normalization and group differential expression for mRNA and the previously obtained miRNA count data was performed with DESeq2 v1.36. Pre-filtering of the dataset was performed to reduce genes that have nearly no information about gene expression by filtering out genes and miRNAs with total counts across all samples less than 10. Count variance stabilization transformation was performed with regularized logarithm transformation (DESeq2 rlog function) and used as input to calculate principal components with plotPCA function from DESeq2 and Euclidean sample distances with dist function from the stats v4.2.2 package. Count normalisation was performed with DESeq function and with log fold change shrinkage. We calculated differential expression in tumour *versus* control and HPV-positive *versus* negative samples (Fig. 1). Differentially expressed genes (DEGs) and miRNA (DEmiR) were selected with BH-adjusted *p* < 0.05 set as significant.

Gene set enrichment analysis (GSEA) was performed and visualised using gProfiler2 v0.2.1 on DEGs using annotated human genes as the background set for the hypergeometric test. We applied the tailor-made multiple test correction method Set Counts and Sizes (SCS)^17^ and a p-value threshold of 0.05. GSEA was performed across multiple databases: Gene Ontology: Biological Processes (GO:BP)^18^, Kyoto Encyclopedia of Genes and Genomes (KEGG)^19^, Reactome^20^, miRTarBase^21^ and TRANSFAC^22^. We used these parameters for all subsequent GSEA.

Identification of miRNA targets was performed by querying significant DEGs against DEmiRs using multiMiR v1.18.0 and the miRTarBase database^23^, selecting only experimentally validated miRNA-gene interactions.

Previously published DNA methylation (DNAm) data^14^ was loaded with RnBeads v2.14.0. Differential methylation (DM) was calculated comparing tumour *versus* control groups on CpG sites and islands (CGIs). Annotation of CpG sites merging into islands was obtained from RnBeads.hg19 v1.28.0 package. All results are presented on CGIs unless otherwise stated. CGI DM was defined as the quotient of methylation between groups across all CpG sites in a CGI, with significance level set to FDR-adjusted *p* < 0.05. Promoter region annotations were obtained from the RnBeads package, defined as 1500 bases upstream and 500 bases downstream of the transcription start site. CGI targets were assigned where CGI overlapped a promoter region.

### Integrative analysis

**Figure 2:**
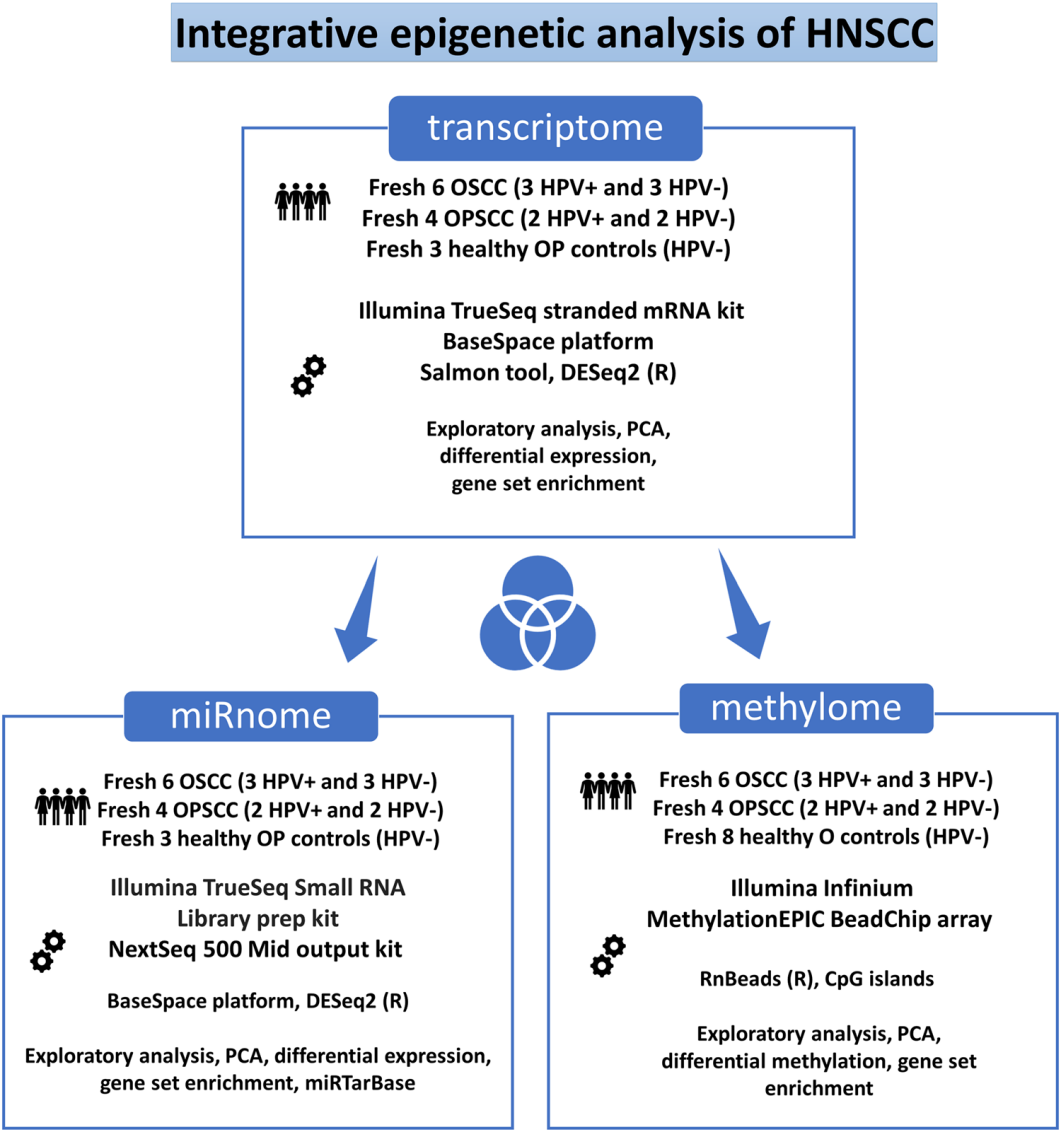
Schematic representation of integrative epigenetic HNSCC study.

Workflow for comparing all omics data and identifying specific mechanisms in HNSCC is summarised in Fig. 2.

Three omics datasets were compared, centred on identified DEGs (Supplemental Table S2). Multiple miRNA-gene pairs were reduced by selecting only the miRNA with highest absolute value of Spearman correlation of counts (mirRNA_Rho) for the given DEG. Multiple gene–CGI pairs were not as common, and the CGI with the lowest p-value was selected for every DEG. Resulting table contained DEGs and their associated miRNA and/or CGI. We assessed the interplay of DEmiRs, DM CGIs and DEGs in two ways: by obtaining simple overlap of DEGs targeted by DEmiRS or DM CGIs and kmeans clustering. The simple overlap was based on whether DEG’s expression levels were associated with DEmiR’s activity and/or CGI methylation levels. Kmeans clustering was performed on log-transformed, centred and scaled values of fold change for DEGs and DEmiRs, and DNAm quotient. We performed kmeans clustering using stats v4.2.2 package, using 8–12 clusters and nstart 100, with the best performing result for 11 clusters.

Survival analysis was performed on all tumour patients using survival v3.5 package, generating survival times, generating Kaplan–Meier plots with default parameters, and testing the difference between the two groups (oral *versus* oropharyngeal location, or HPV-positive *versus* HPV-negative samples) using the default G-rho test.

### Validation against TCGA datasets

The HNSCC dataset was obtained from the TCGA repository (https://portal.gdc.cancer.gov) specifying tumour site as tonsil, floor of mouth, tongue and oral cavity. The transcriptome, and miRNome data were imported as raw gene counts and analysed using DESeq2. Methylome data were imported as beta values and analysed using limma v3.46. The further analysis focused on the 1834 genes found to be significantly differentially expressed in both our cohort and the TCGA dataset, as presented in Fig. 7a.

### Ethical approval and consent to participate

All patients provided informed consent to participate at the time of primary cancer treatment. The collection of samples was approved by Bioethical Bord of the Ruđer Bošković Institute (BEP-3748/2-2014) and the Ethical Board of the Clinical Hospital Dubrava (EP-KBD-10.06.2014). The research was performed in accordance with relevant guidelines and regulations including the Declaration of Helsinki.

## Results

### Transcriptome profiling

**Figure 3:**
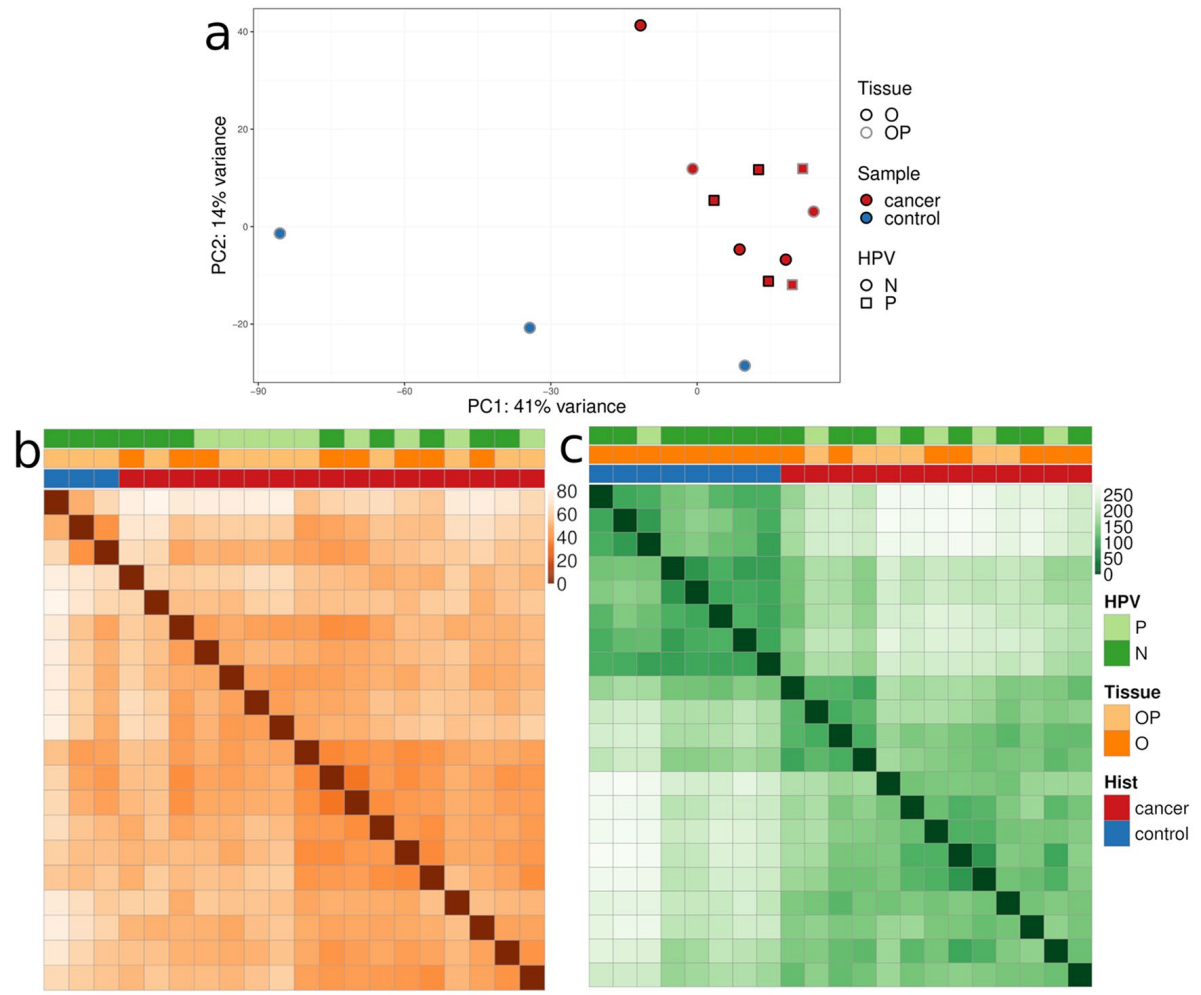
Exploratory analysis of three omics datasets: (**a**) PCA plot for transcriptome data; (**b**) sample distance heatmap for miRNome data; (**c**) sample distance heatmap for methylome. N, HPV-negative; P, HPV-positive; O, oral tumours; OP, oropharyngeal tumours*.*

The whole transcriptome profiling was performed on tissue samples from 13 individuals with different anatomical locations and HPV infection status (Fig. 1). The top two principal components (PCs) of the transcriptome (Fig. 3a), which explain 55% of the variance in the samples, splits samples into two clusters of tumour and control samples. No obvious contribution of HPV status or tumour anatomical location was observed in any of the top 10 PCs, totalling to 84.4% variance explained (Supplemental Fig. S3a). We observe the same pattern of dominant differentiating factor being tumour *versus* control status in miRNome and methylome data (Fig. 3b,c), and the lack of obvious pattern for tumour anatomical location or HPV status (Supplemental Fig. S3b), based on the Euclidian distance of sample profiles. Finally, survival rates showed no significant differences between the HPV-positive and negative patients, nor between the oral and oropharyngeal tumour locations (Supplemental Fig. S4).

### Differential expression and GSEA

**Figure 4:**
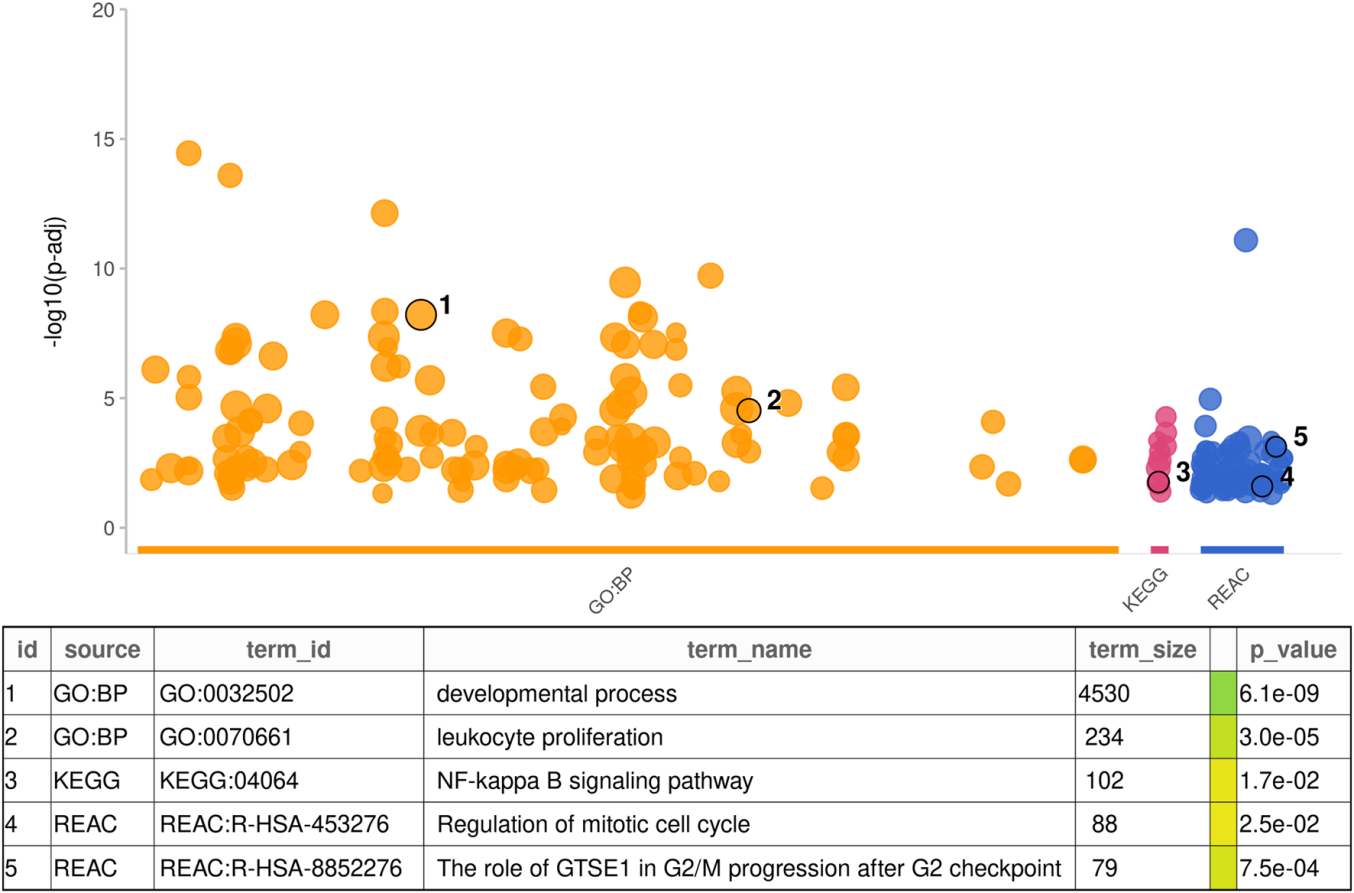
Gene enrichment analysis of tumour *vs.* control samples. Databases shown are GO:BP (orange), KEGG (pink), and REACTOME (REAC, blue). The highlighted terms correspond to key concepts associated with cancer progression and regulation.

Exactly 18,104 genes had no less than 10 detected reads across samples. Differential expression analysis revealed that 1248 (6.9%) of genes were significantly overexpressed and 1533 (8.5%) were significantly underexpressed in tumour (BH-adjusted *p* < 0.05), when compared to transcriptomes of control samples. GSEA on DEGs showed enrichment of signalling pathways associated with tumour progression, such as cell cycle regulation and oncogenic signalling pathways such as NF-kB (Fig. 4); for a full list of terms see Supplemental Table S3.

### Differential miRNA and DNA methylation assessment

Integration of 514 significant DEmiRs based on MiRNome data^13^ with the database containing experimentally supported miRNA-target gene interactions yielded 3042 interactions among 90 different miRNAs and 1227 target genes, hereafter termed DEmiR-DEGs. In our analysis most of these genes were regulated by a single miRNA, however, some were associated with multiple miRNAs. Notably, two genes with the highest number of associated DEmiRs, *BCL2* and *MYC*, which play a role in regulating cell proliferation and apoptosis, were associated with 18 and 16 miRNAs, respectively. In addition, some miRNAs were associated with many target genes, harbouring potential therapeutic value by potentially affecting many cancer-related signalling pathways. An extreme example is a frequently reported tumour suppressor hsa-miR-26b-5p putatively forming interactions with 255 unique DEGs. Within our dataset hsa-miR-26b-5p showed a high negative expression correlation with known oncogenes such as *MMP10* (Rho = − 0.81, *p* < 0.005), *IGSF3* (Rho = − 0.79, *p* < 0.005) and *ARNTL2* (Rho = − 0.79, *p* < 0.005) (Supplemental Table S2).

Overall methylation levels in our samples showed that more than 300,000 CpG sites were hypomethylated in tumour samples compared to controls (FDR-adjusted *p* < 0.05), while 72,000 were hypermethylated (Supplemental Table S4). We also noted a larger number of hypomethylated CGIs (6557) than hypermethylated (5457), although the difference is not as pronounced as in CpG sites (Supplemental Table S5). The DM CpGs predominantly fell into gene bodies, but also some fell in intergenic regions corresponding to repeated elements. These regions consistently showed a larger number of hypomethylated CpGs compared to hypermethylated ones (Supplemental Fig. S1).

To identify deregulated tumour suppressors in our samples, we used the TSGene database 2.0^24^ containing tumour suppressors curated from several thousand publications combined with expression and mutational profiles. We pinpointed 10 genes that were underexpressed and contained hypermethylated CGIs in the promoter region in our samples (Supplemental Table S6).

To assess the effects of transcription factors on DEGs that had differentially methylated CGIs (DM-DEGS) within our dataset we performed GSEA using transcription factor database TRANSFAC. We found associations with several groups of transcription factors including *E2F*, *SP/KLF* and *AP-2* families (Supplemental Table S7). 

Of note we found the enrichment of genes regulated by the *E2F* family of TFs (SCS-adjusted *p* < 10^–43^), which play a critical role in cell cycle control. Furthermore, GSEA on the same dataset using the Reactome database showed enrichment of genes involved in cell cycle regulation such as G1/S Transition and G2/M Checkpoints (SCS-adjusted *p* < 10^–4^), supporting the above findings.

### Integrative analysis: influence of miRNome and methylome on transcriptional activity in HNSCC

**Figure 5:**
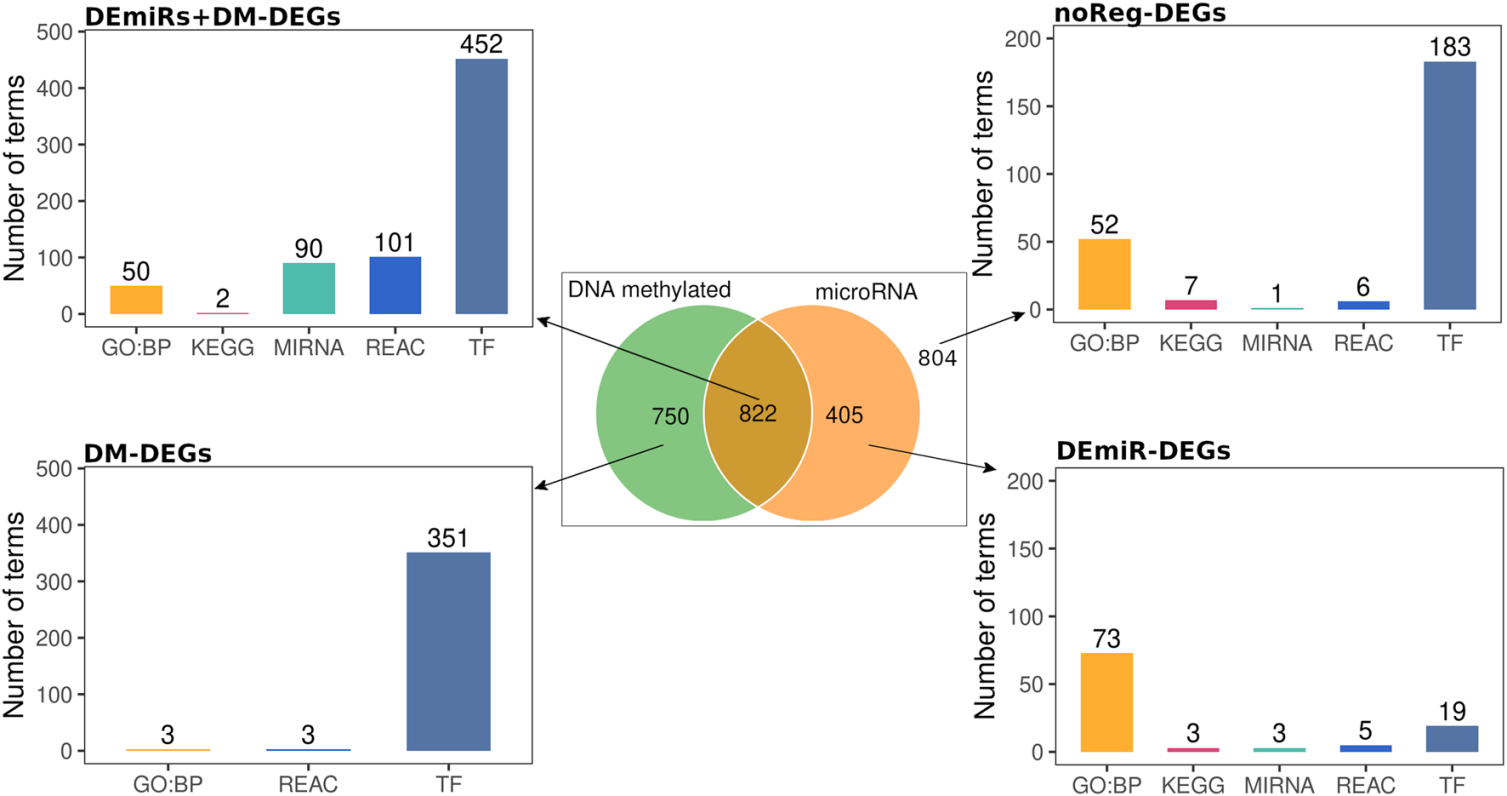
DEGs between tumour and control samples, in association with DEmiR or DM promoters. Four panels show number of terms from GSEA for each group.

Each DEG was grouped based on the association with DEmiR or DM CGI, shown in Fig. 5. We obtained four groups: DEGs associated only with DEmiR (DEmiR-DEGs), DEGs associated only with DM CGIs (DM-DEGs), DEGs associated with both DEmiR and DM (DEmiR + DM-DEGs), DEGs not associated with either DEmiR or DM CGIs (noReg-DEGs). The groups had different proportion of differentially represented GSEA terms, i.e. GO:BP, KEGG, miRTarBase (MIRNA), Reactome and TRANSFAC (TF) (Fig. 5, Supplemental Table S8). The DM-DEGs group was enriched by genes regulated by TFs but no significant results of GO biological pathways were detected. The DEmiR-DEGs group was enriched with pathways related to signalling and immunological systems. The noReg-DEGs group was enriched with immunological processes and muscle system processes. DEmiR + DM-DEGs were enriched with general regulatory processes: biological, metabolic and cellular processes. These terms are high level terms in GO biological processes, suggesting that genes in the intersection have broad regulatory roles.

Clustering of DEGs with accompanying DEmiR and DM levels revealed a critical distinction in the epigenetic factors driving HNSCC. The identified clusters predominantly demonstrated either significant DEmiRs or DM. This suggests that in HNSCC, gene expression is predominantly influenced by either miRNA regulation or methylation, but not both concurrently. This key finding illuminates the mutually exclusive roles of miRNA and methylation in shaping the gene expression landscape in HNSCC.

It is generally believed that miRNA and DNAm have an inhibitory effect on gene expression, and this pattern was evident in clusters 3, 4, 7 and 10. In addition, we observed miRNA expression and methylation patterns in clusters 1, 5, 6, and 9 that were indicative of noncanonical regulatory activity. It is important to mention that due to multiple target genes of miRNAs there was a significant overlap of underexpressed miRNAs in clusters 3 and 9 (73%) and overexpressed miRNAs in clusters 5 and 7 (77%).

**Figure 6:**
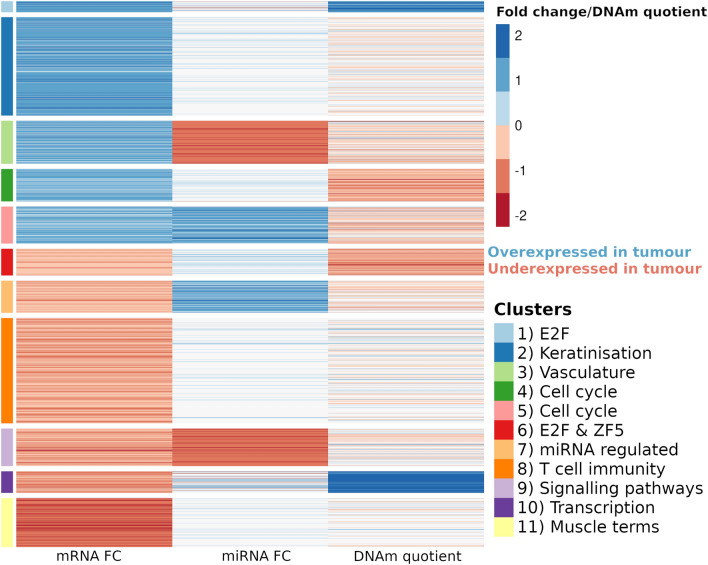
Kmeans clustering on integrated transcriptome, miRNome and methylome data on DEGs. Fold change values for transcriptome and miRNome and DNAm quotient were scaled and normalised prior to clustering. Eleven clusters are functionally described by applying GSEA over several databases (GO:BP, KEGG, REACTOME, MIRNA and TRANSFAC).

GSEA analysis of each cluster linked expression and epigenetic patterns to biological function. Representative terms that best describe each cluster are shown in Fig. 6 and the full GSEA results for each cluster are shown in Supplemental Table S9. DM-DEGs in clusters 1, 4, 6 and 10 were highly enriched with genes regulated by the *E2F* family of TFs. Most of the genes from the *E2F* family were not differentially expressed in our samples (Supplemental Fig. S2), although it has been previously shown that the methylation status of CpG sites of* E2F* binding motif influences the binding of the transcription factor^25,26^. In addition, cluster 6 contained underexpressed genes with hypomethylated CGIs and was enriched with genes regulated by the *ZF5* transcription factor, which can function as a repressor^27^. Clusters 4 and 5 were associated with similar terms related to various cell cycle processes but have different patterns of miRNA expression and DNAm.

**Figure 7:**
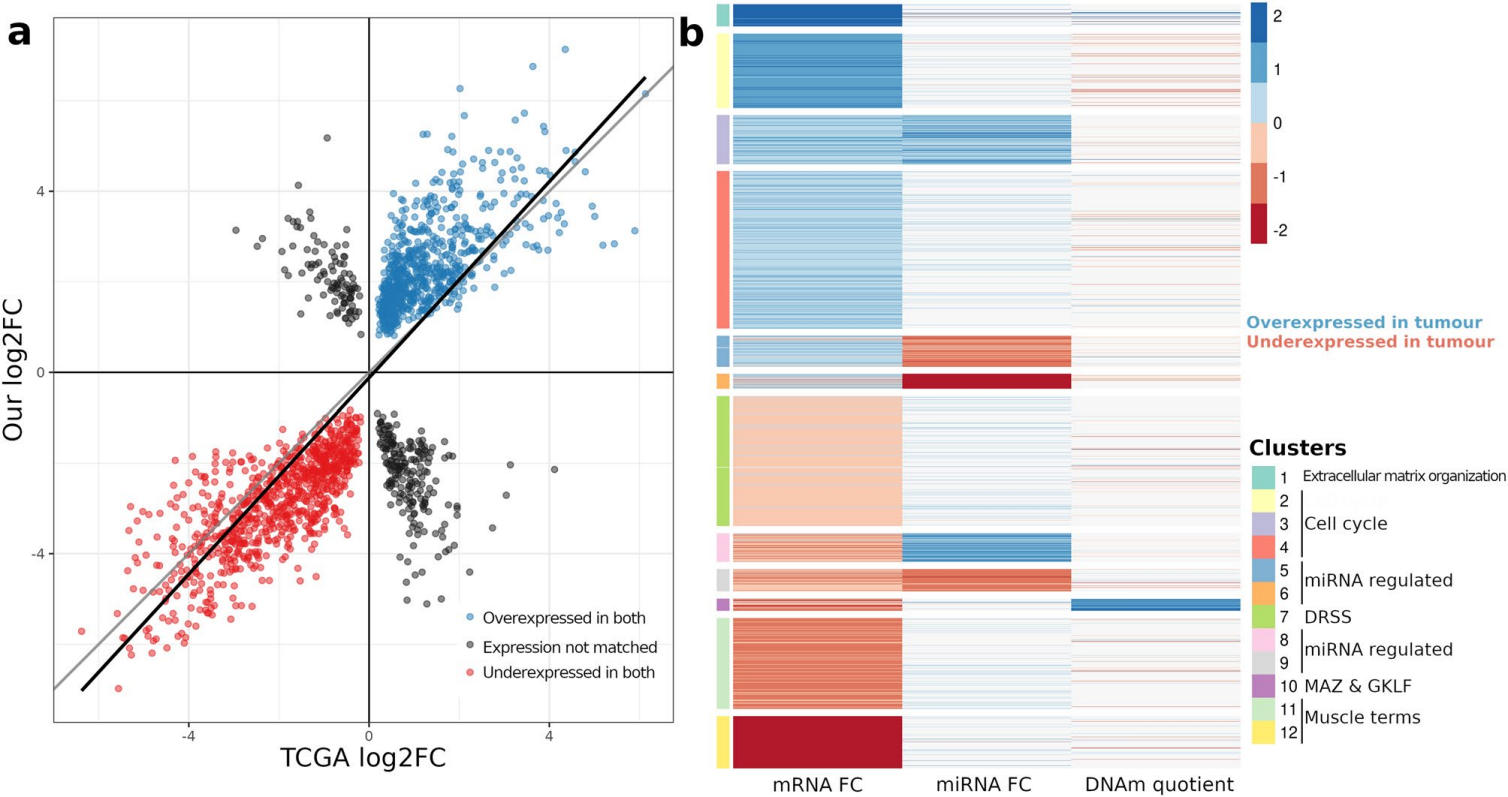
TCGA validation: (**a**) fold change values of 1834 DEGs found both in our dataset and TCGA. Grey line shows y = x identity line and black line shows line of best fit though presented data; (**b**) kmeans clustering on integrated transcriptome, miRNome and methylome TCGA data on 1834 DEGs.

To validate results obtained in our cohort, we have analysed TCGA HNSCC transcriptomes, methylomes and miRNomes as similarly as we could, based on the provided experimental details (Supplemental Tables S10, 11). The validation TCGA dataset had shown the same exclusivity in employment of either methylation or miRNA regulation of DEGs (Fig. 7b), with high correlation of DEG profiles in our cancer *versus* control analysis, when compared to TCGA expression changes of the same genes (Fig. 7a)(Pearson correlation coefficient of 0.76; *p* < 2.2 × 10^–16^).

### Influence of HPV

**Figure 8:**
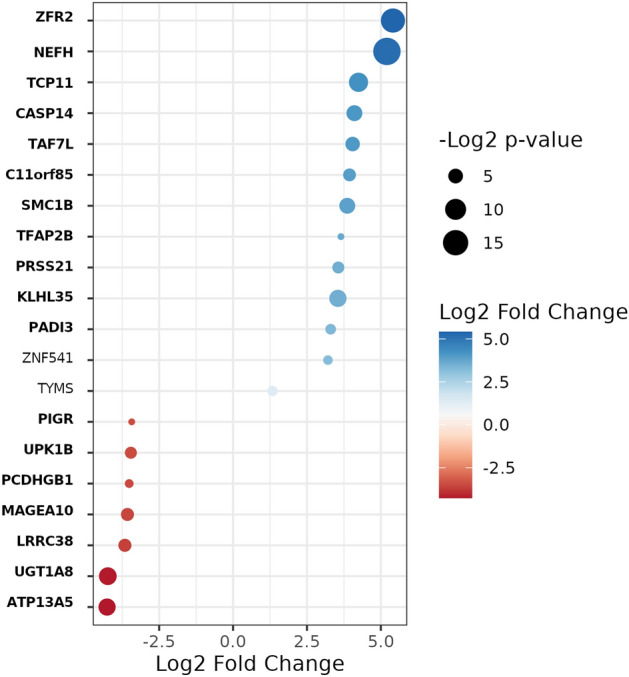
DEGs in HPV-positive compared to HPV-negative tumour patients, in this study (shown in bold) or previously found to be related to HPV-associated cancers.

HPV influence on gene expression was found to be inconclusive (Fig. 3). We observed a small number of genes and miRNAs, and no DM CGIs or sites that were significantly changed while comparing HPV-positive and negative tumour sample groups (FDR-adjusted *p* < 0.1). Number of DEGs associated with HPV status was 44, of which 25 were overexpressed (Supplemental Tables S12, 13). Among these, we focused on the selection of DEGs, specifically top 18 genes based on the absolute log2FoldChange value, and additional 2 genes previously identified as biologically relevant to HPV status in HNSCCs^28,29^ (Fig. 8).

## Discussion

In this study, we conducted an integrative analysis of miRNome, methylome and transcriptome data to unravel regulatory processes driving the development and progression in HNSCC. Using unsupervised clustering, we revealed distinct patterns in the three omics datasets, suggesting that the influence of miRNAs and DNAm on gene expression regulation is mostly exclusive. Clustering also revealed key pathways associated with tumour progression including dysregulation of cell cycle processes, transcription, immune response modulation, and vasculature-related genes (Fig. 6). Furthermore, we highlight the importance of the DNAm in promoter regions of genes found to be targets of the *E2F* family.

Our transcriptome analysis divided samples into two major clusters: tumour and control samples, with no obvious contribution of HPV status or tumour anatomical location. Similar results were seen in the miRNA expression profile study and DNAm study in an overlapping set of samples (Fig. 3b,c) as previously published^13,14^. These findings suggest that tumour profiles presented here may share similar cells of origin or common pathways during tumourigenesis. The emergence of tumour progression pathways among DEGs (n = 2781, Fig. 4) resembles transcriptomes of radioresistant HNSCCs, where hypoxia response, p53 pathway, NF-kB pathway and inflammatory response were found abnormally activated^30^.

The clustering presented in Fig. 6 indicates that distinct regulatory mechanisms influence the gene expression patterns in tumours compared to control samples, with specific biological processes enriched for some clusters. Two clusters of overexpressed genes which are involved in the key cell cycle regulation pathways have mostly hypomethylated promoters. Furthermore, among underexpressed genes in tumours, we observed a cluster of DM-DEGs related to regulation of transcription (Fig. 6, cluster 10). Clusters 4 and 10 suggest a common influence of DNAm on gene expression regulation, where methylation of gene promoters inhibits expression and a lack of methylation promotes gene expression^31–33^. However, a reverse trend of overexpression + hypermethylation and underexpression + hypomethylation appeared in clusters 1 and 6 respectively, both enriched with *E2F* and other transcription factor targets. Similarly, an inhibitory effect of miRNAs on target genes are shown in cluster 3 enriched with vasculature related genes and cluster 7 with no specific biological processes detected, except for grouping of miRNA targets.

A positive correlation of miRNAs and target genes are detected in clusters 5 and 7, as previously observed in multiple studies^34,35^. While our results could imply either a direct or indirect effect of miRNA on DEmiR-DEGs potentially through negating the action of repressive miRNA protein complexes^34^, it is important to note that our findings represent correlation, not causation. Further studies are required to establish the causal mechanisms involved. In addition, it is worth considering that miRNAs often have multiple targets, and the interactions in existing databases may be derived from high-throughput methods that may not reflect the full complexity of in vivo biological interactions. Finally, clusters 2, 8 and 11 show no strong patterns of regulation by miRNA or DNA. Of note, cluster 11 consisted of genes involved in muscle system processes, a result which was replicated in the TCGA data (Fig. 7b).

Interestingly, we found a cluster of genes under control of the transcription factor *E2F*, that were significantly underexpressed in tumour samples in comparison to controls (Fig. 6, cluster 6). Previous studies show that the* E2F* family of genes are (negatively) regulated by miRNAs in HNSCC^36^ as well as other cancers such as lung cancers^37^, paediatric brain tumours^38^, and chronic myeloid leukaemia^39^. Furthermore, the TCGA study found an increase in copy numbers of the *E2F1* gene in the HPV-positive HNSCCs^36^. *E2F *is often highly expressed and plays a fundamental role in regulating tumour growth and proliferation, metastasis and drug resistance in cancer^40^. Interestingly, our study did not find *E2F* family to be overexpressed in cancer with respect to controls (Supplemental Fig. S2), however clustering revealed multiple dysregulated gene clusters which correspond to targets of the E2F family, i.e. clusters 1, 4, 5, 6 and 10. The most prominent ones are clusters 4, 5 and 10 which are involved in cell cycle and transcriptional regulation that also exhibit differentially methylation in promoter regions (Supplemental Table S7 and Fig. 6). *E2F1* activation has previously been linked with the methylation status of *HOX* genes in oral cancer^40^, and our findings expand this to multiple pathways involving cell cycle control.

The importance of immune modulation in HNSCC progression can be investigated in cluster 8, enriched with genes related to T cell immunity and demonstrating underexpression in tumour samples, targeted by overexpressed miRNAs. This dysregulation of T cell immunity genes is to a lesser extent associated with hypermethylation, which reflects the complex interplay between immune response regulation and the tumour microenvironment, emphasizing the role of immune evasion mechanisms in HNSCC. Indeed, we found increased hypomethylation in immune system genes is in line with trends detected in several previous methylation studies of head and neck cancers^41,42^, and in cervical cancers^43,44^. T cell exhaustion is largely conserved in cancer cells and has previously been related to epigenetic regulation of tumour progression^45^. However, given that the controls for this integrative study were derived from the inherently immunologically active tonsil tissue, the findings related to cluster 8 should be interpreted with caution.

Our results showed that genes involved in vascularization in tumours are DEmiR + DM-DEGs, likely influenced by both epigenetic drivers, with a dominant effect of DEmiRs (Fig. 6, cluster 3). Moreover, other authors found a link with DNAm and cardiovascular diseases directly rather than changes driven by miRNA expression^46^, or that both epigenetic changes are involved in vascular diseases, claiming that, the most prominent gene cluster is activated via hypomethylation^47^. Previous studies show that downregulation of miRNAs lead to overexpression of genes involved in angiogenesis^48^. For example, miR-150 which is underexpressed and targets multiple genes in cluster 3 (Supplemental Table S2), is found to be a tumour suppressor in colorectal cancer^48^. Furthermore, the extracellular matrix plays a crucial role in modulating angiogenesis^49^. In cluster 3 we found overexpression of several matrix metallopeptidase genes (*MMP2*, *MMP10*, *MMP11* and *MMP13*) which are also reported upregulated in other cancer types^50^. Matrix metallopeptidase genes are common targets of miRNA, and the dysregulation of the interactions are often observed in cancer^51,52^. We therefore demonstrate the potential role of both epigenetic modalities in HNSCC by modulating vasculature-related processes, with dominating DEmiRs as epigenetic silencers. The effect of the HPV status of a tumour was investigated on a small number of genes found significantly differentially expressed in HPV-positive *versus* HPV-negative tumours in our study (Fig. 8). There has been previous evidence of differential expression of e.g. *NEFH*, *ZRF2,* *TAF7L*, *ZNF541* and *TYMS* in head and neck cancer, related to HPV status^28,29,53–55^. Notably, some authors have already found hypermethylation of *NEFH* (protein kinase binding and microtubule binding), in pharyngeal squamous cell carcinoma, which significantly correlated with HPV positivity^53^. *ZFR2* gene codes for RNA-binding protein characterized by its domain associated with zinc fingers and zinc ions. It was found overexpressed in HPV‐positive HNSCC patients, when compared to HPV‐negative patients, and has been flagged as a prognostic for the cervical cancer cases^54^. The expression of the transcription factor *TAF7L* (TAF7-like RNA polymerase II) has been already found increased, even 220-fold, in HPV-positive HNSCCs^55^. *ZNF541* encodes a zinc finger protein supposed to be a component of chromatin remodelling complexes. The hypomethylation and higher expression of this gene was also observed in HPV-positive oropharyngeal squamous cell carcinoma (OPSCC) and significantly associated with a better overall survival, independent of HPV status^28^. *TYMS* gene codes for thymidylate synthetase that catalyses the methylation of deoxyuridylate to deoxythymidylate. In HPV-positive cancers they have found it overexpressed, indicating that HPV-positive oropharyngeal cancers may be more resistant to 5-fluorouracil chemotherapy than HPV-negative^29^.

We presented a comprehensive study on an overlapping set of samples, where the integration of miRNome, methylome and transcriptome analyses in HNSCC was conducted. We acknowledge certain limitations of our study, including the relatively small sample size, which may affect the validity of our findings. In addition, obtaining control samples that are genuinely representative of healthy tissue poses its own set of challenges. However, our findings were further validated on a much larger dataset from the TCGA programme. Associating miRNome and methylome with transcriptome profiles, we found many DEGs in HNSCC in comparison to control samples, that were mainly associated with the cell cycle regulation, transcription, immunity and tumour development. We demonstrated the majority of genes belong to clusters associated exclusively with miRNome or methylome and, to a lesser extent, under the control of both epigenetic mechanisms.

### Supplementary Information

Supplementary Figures.

Supplementary Tables.

## Data Availability

Code used is available at https://github.com/mlkr-rbi/HNSCC_Integrative_analysis. All datasets and raw data are deposited in ArrayExpress repository, accession numbers: transcriptome E-MTAB-13725, miRNome E-MTAB-13953 and methylome E-MTAB-13954. Due to the file size restrictions Supplementary Table S4 is deposited at Figshare repository https://doi.org/10.6084/m9.figshare.24722268.
