# Supplementary material for: Integrative analysis in head and neck cancer reveals distinct role of miRNome and methylome as tumour epigenetic drivers
Source: Sci Rep. 2024 Apr 20;14:9062. doi: 10.1038/s41598-024-59312-z (PMC11032388; doi:10.1038/s41598-024-59312-z)
Supplement: Supplementary file 1 — Supplementary Figures. [file 41598_2024_59312_MOESM1_ESM.pdf]

Supplemental figures

Integrative analysis in head and neck cancer reveals distinct role of miRNome and methylome as tumour epigenetic drivers

Katarina Mandić[1]*, Nina Milutin Gašperov[2]*, Ksenija Božinović[2], Emil Dediol[3], Jure Krasić[4], Nino Sinčić[4,5], Magdalena Grce[2], Anja Barešić[1]**, Ivan Sabol[2]**

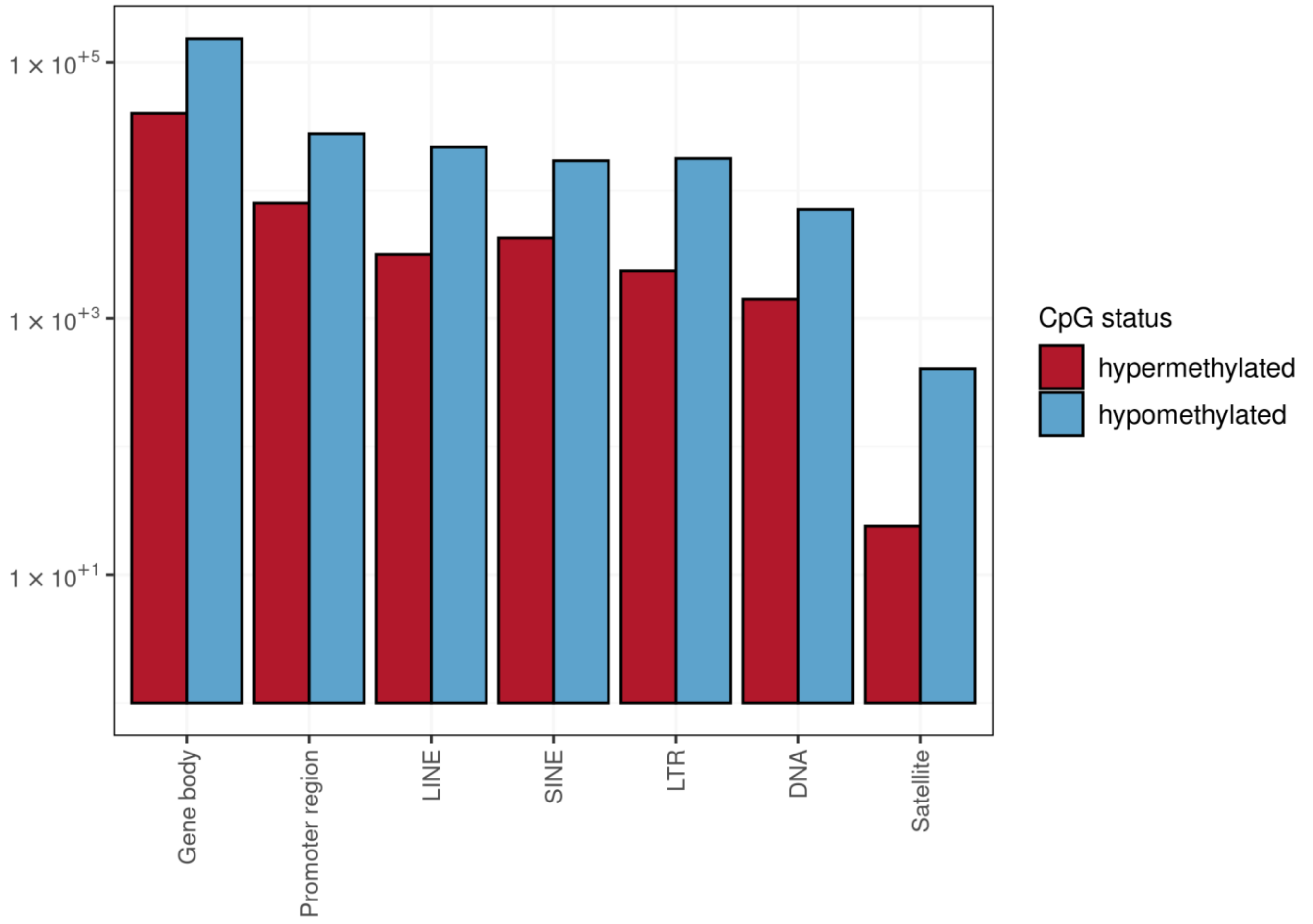

Suplemental figure 1. Significant number of hypermethylated and hypomethylated CpGs in cancer vs. control groups across all genomic elements (Chi squared p < 2x10^-16). Labels: Gene body – across exons and introns; Promoter region – from 500 to 2000 distance from canonical transcription start site; LINE – Long interspersed nuclear elements; SINE – Short interspersed nuclear elements; LTR – Long terminal repeat elements which include retroposons; DNA – DNA repeat elements; Satellite – Satellite repeats. Annotation for genomic elements were used from Jin et al., 2015.

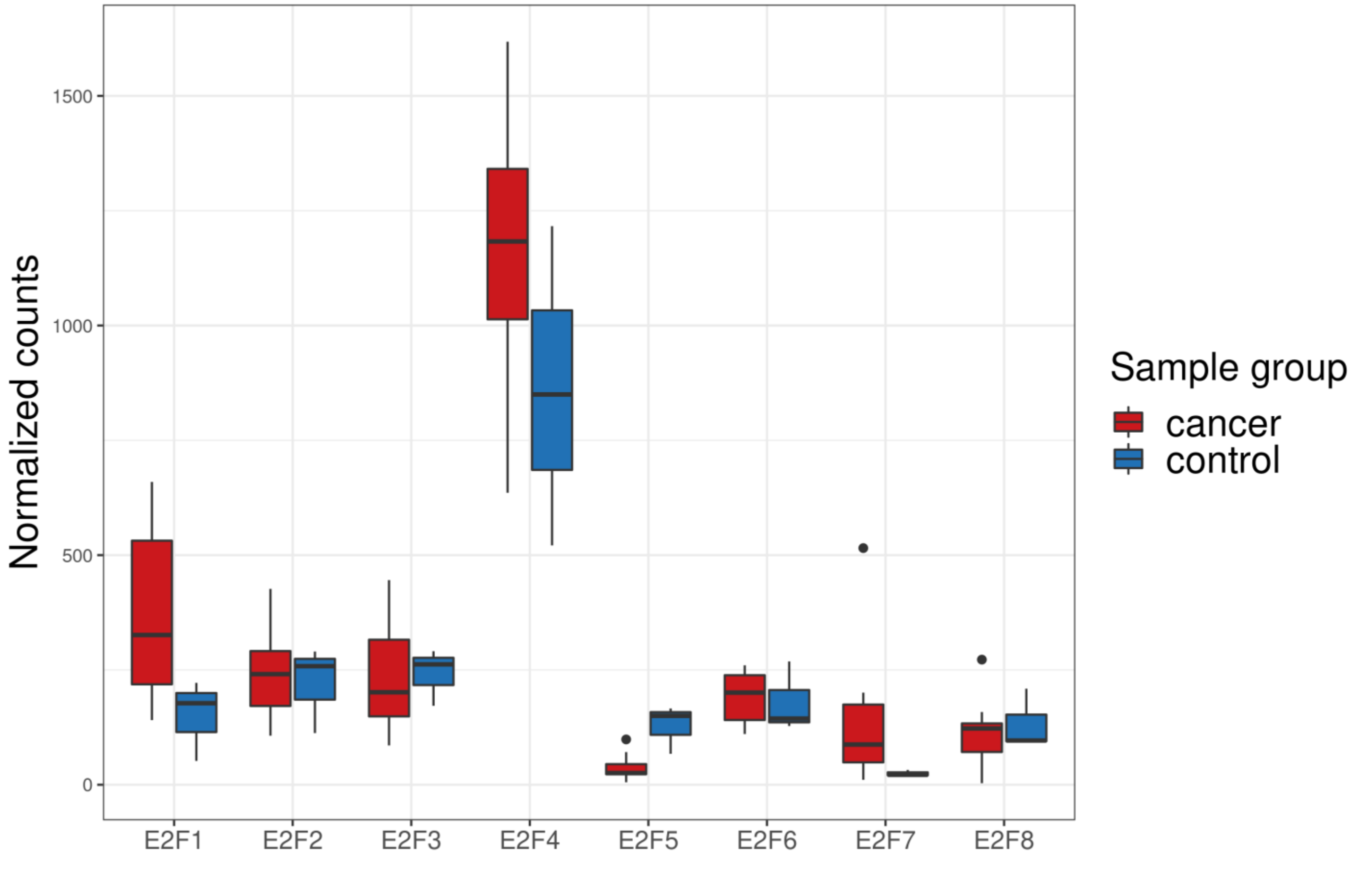

Supplemental figure 2. Expression of E2F family genes in cancer vs control samples.

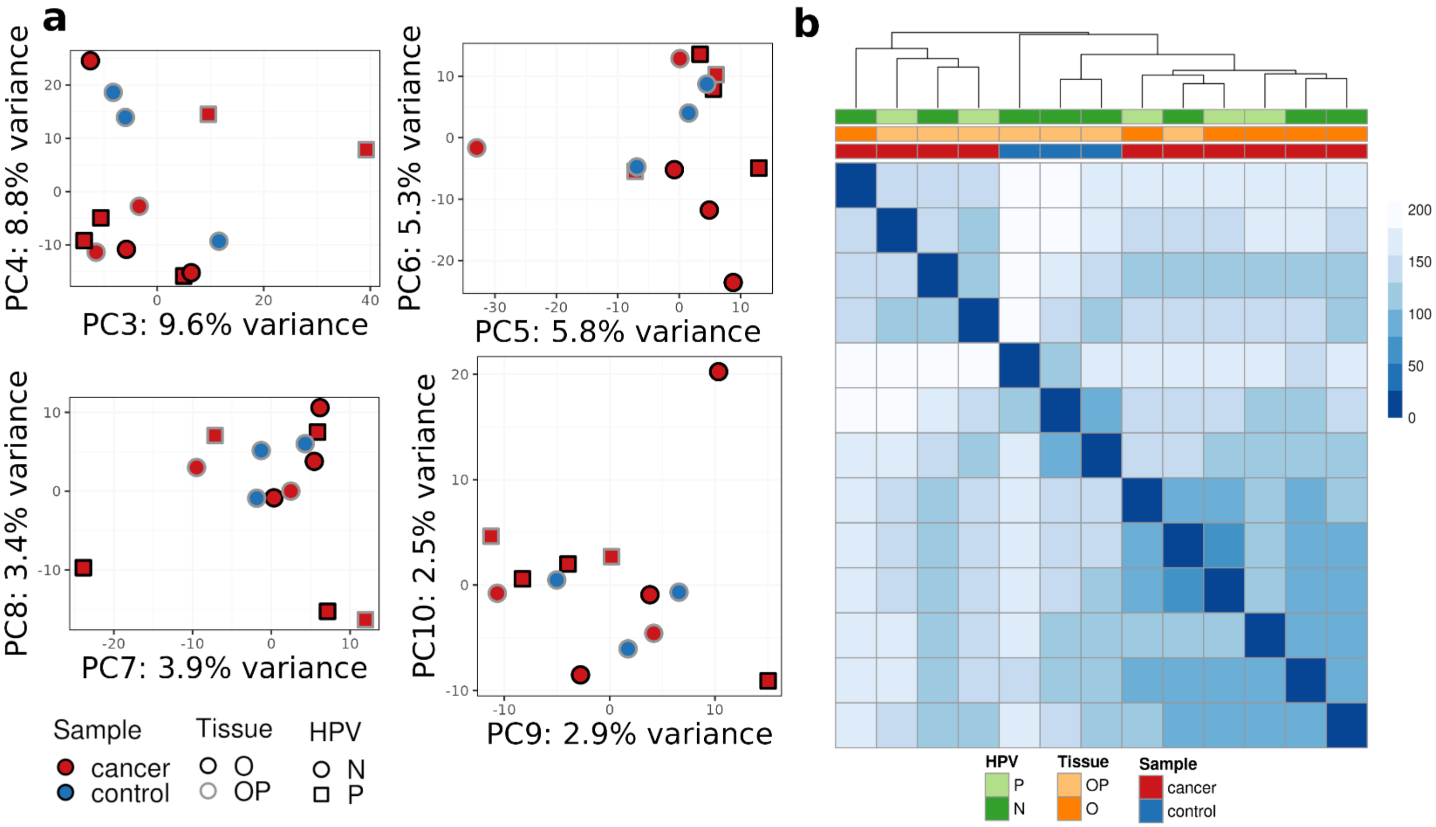

Supplemental figure 3. Dimensionality reduction on transcriptome data: a) Principal components 3-10; b) Sample distance heatmap using Euclidian metric

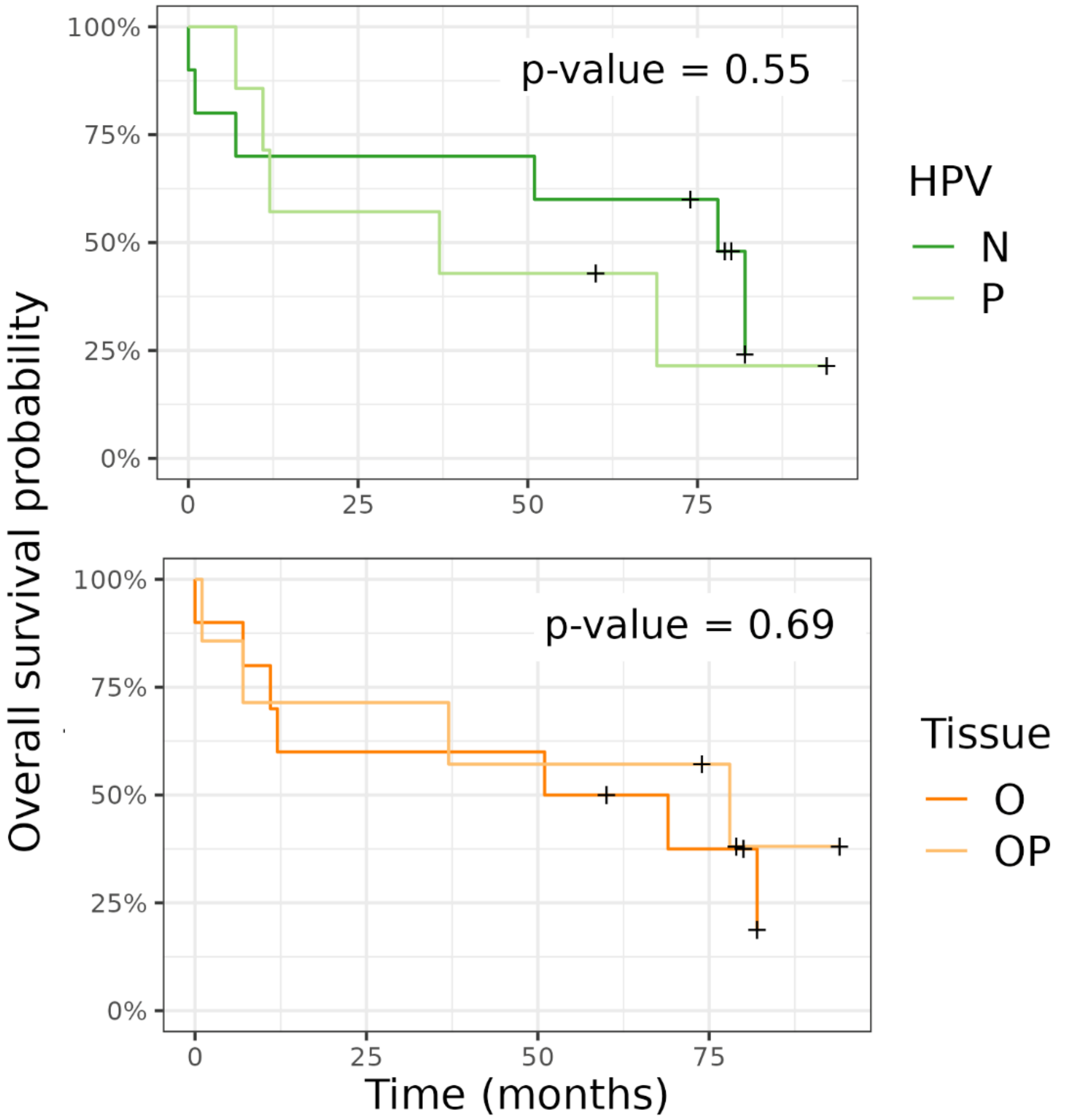

Supplemental figure 4. Kaplan-Meier plots showing survival rates in HPV positive versus negative patients and based on tumour location oral versus oropharyngeal.
